# Preparation and Characterization of the Forward Osmosis Membrane Modified by MXene Nano-Sheets

**DOI:** 10.3390/membranes12020146

**Published:** 2022-01-25

**Authors:** Yuqi Nie, Chaoxin Xie, Yi Wang

**Affiliations:** 1 Department of Military Installation, Army Logistics Academy of PLA, Chongqing 401331, China; nyq603394605@sina.com (Y.N.); a86909304@163.com (C.X.); 2 State Key Lab of NBC Protection for Civilian, Beijing 102205, China

**Keywords:** forward osmosis membrane, MXene nano-sheets, water flux, reverse solute flux

## Abstract

The Forward Osmosis (FO) membrane was the core of FO technology. Obtaining a high water flux while maintaining a low reverse solute flux has historically been considered the gold standard for a perfect FO membrane. In a thin-film composite FO membrane, the performance of the membrane was determined not only by the material and structure of the porous support layer but also by the structural and chemical properties of the active selective layer. Researchers have selected numerous sorts of materials for the FO membranes in recent years and have produced exceptional achievements. Herein, the performance of the modified FO membrane constructed by introducing new two-dimensional nanomaterial MXene nano-sheets to the interfacial polymerization process was investigated, and the performance of these modified membranes was investigated using a variety of characterization and testing methods. The results revealed that the MXene nano-sheets played an important role in improving the performance of the FO membrane. Because of the hydrophilic features of the MXene nano-sheets, the membrane structure may be tuned within a specific concentration range, and the performance of the modified FO membrane has been significantly enhanced accordingly. The optimal membrane water flux was boosted by around 80%, while its reverse solute flux was kept to a minimum of the resultant membranes. It showed that the addition of MXene nanosheets to the active selective layer could improve the performance of the FO membrane, and this method showed promising application prospects.

## 1. Introduction

To cope with the shortage of freshwater resources, people began to develop different types of water treatment technologies to solve such kinds of problems [1]. The membrane water treatment technology is one of the water treatment technologies that has been widely used and has achieved remarkable results in drinking water purification, wastewater recovery and seawater desalination [2]. The membrane water treatment technology mainly includes nanofiltration [3], microfiltration [4], ultrafiltration [5], reverse osmosis [6], etc. At present, reverse osmosis is one of the most widely used and most efficient technologies. However, because of the significant energy consumption and severe fouling issues involved in the process of its utilization, it has always been regarded as a bottleneck, prompting people to search for breakthroughs on a regular basis. Thus, the forward osmosis (FO) technology has demonstrated tremendous development potential due to its low energy consumption and less membrane fouling. This technology showed the potential to revolutionize the water treatment industry [7], which quickly attracted the wide interest of lots of researchers. However, because of the presence of concentration polarization [8], which was one of the primary factors limiting the improvement of FO technology, the actual result of FO membrane water flux was significantly lower than the theoretical value, which severely restricted the FO technology’s widespread usage and application. 

In this context, researchers began to explore new raw materials on a continual basis in the hope of compensating for the shortcomings of FO membranes by exploiting the properties of certain materials. So far, a huge number of new materials have been developed by human beings, which are suitable for preparing FO membranes and can significantly improve their performance [9], such as TiO_2_ [10], graphene [11], carbon nanotube [12], and other materials. In comparison with the original membrane, the modified membrane prepared by these materials showed improved hydrophilicity, thus alleviating the concentration polarization phenomenon and greatly improving the water flux in the meantime, promoting the development of FO technology. In recent years, two-dimensional transition metal carbide MXene nano-sheets [13] have gradually entered people’s vision. Compared with the previously widely used nano materials, MXene nano-sheets are two-dimensional flake nano fillers with a high specific surface area, adjustable layer spacing, excellent chemical stability, and good thermal resistance [14], and have shown good application performance in other separation membrane fields [15]. For example, Wang et al. used double-layer MXene nano-sheets as rigid components to prepare the support membrane by vacuum-assisted filtration, and it was found that the synthetic membrane had highly ordered nanochannels and only allowed specific molecules to pass through, and its penetration into water and organic matter reached an amazing 2300 and 5000 LMH bar^−1^, respectively [16]. Pandey et al. prepared the modified nanofiltration membrane by blending Ti_3_C_2_Tx with cellulose acetate in the phase conversion process. The results showed that the water flux of the composite membrane was as high as 256.85 LMH bar^−1^ [17]. In the research field of FO membranes, Alfahel et al. prepared a modified FO membrane by blending MXene and cellulose acetate (CA) by the phase conversion method, which aimed to study the FO membrane application in seawater desalination and wastewater treatment. It was found that the addition of MXene improved the membrane water flux and reverse solute flux at the same time, and the MXene modified membrane was better than the commercial membrane in anti-fouling ability [18]. Xing Wu et al. investigated the construction of an MXene intermediate layer between the nylon matrix and the PA layer and also studied the performance of the as-prepared membranes by adjusting the concentration of MXene and brushing times. The results showed that this method broke the well-known trade-off effect, increased membrane water flux while decreasing reverse solute flux, as well as significantly improved the permeability of ethanol [19]. It can be seen that the MXene nano-sheets had great potential in the direction of water treatment, but so far, there had been few systematic research on its application in the field of the thin-film composite FO membrane, which still demanded further exploration. At present, the thin-film composite FO membrane prepared by interfacial polymerization technology has been widely studied and applied because it can be flexibly regulated according to the requirements of membrane performance and has a wide range of applications [20]. Generally, the active selective layer is formed at the top of the porous support layer through interfacial polymerization. Therefore, the physical structure state and chemical element composition of the active selective layer play an important role in the separation efficiency of the FO membrane [21,22,23].

In this study, a new two-dimensional hydrophilic nanomaterial, MXene nano-sheets, were implanted into the aqueous phase in the interfacial polymerization process to prepare a new nano-modified thin-film composite FO membrane with different addition amounts of MXene nano-sheet, and a series of characterizations and analyses were carried out on the aspects of surface structure, physical properties, chemical composition, and permeability. After optimization and experimental analysis, the FO membrane with high separation performance was finally obtained. This study showed a good and promising protocol for MXene-modified FO membrane fabrication and synthesis. 

## 2. Materials and Methods

### 2.1. Experimental Materials

Polysulfone (PSf, average molecular weight of 22,000da) and trimethyl chloride (TMC, >98%) were purchased from Sigma-Aldrich, (St. Louis, MO, USA). Polyvinylpyrrolidone (PVP, K30), n-hexane (chromatographic grade, >98%), m-phenylenediamine (MPD, analytical purity >99.5%), and N-methyl pyrrolidone (NMP, analytical purity >99%) were purchased from Aladdin, Shanghai, China. From Beike 2D Materials Co., Suzhou, China, MXene nano-sheets (single layer) were obtained. Sodium chloride was purchased from Macklin, Shanghai, China. Pure water (conductivity less than 5 μs/cm) was self-made. 

### 2.2. The Preparation of the MXene Nano-Sheets Modified FO Membrane

#### 2.2.1. The Preparation of the Porous Support Layer

**Figure 1:**
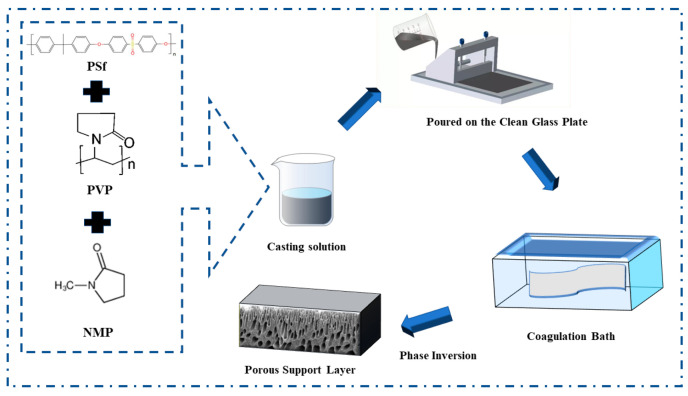
The porous support layer preparation by the phase inversion method.

At a room temperature of 25 °C, the PSf, PVP, and NMP were mixed in the container in a 7:2:40 ratio for 24 h, and the resulting mixture was degassed for 12 h to produce a clear, homogeneous casting solution. The resulting mixture was scraped with the casting solution evenly on a clean glass plate, then placed in a water bath for phase conversion to obtain the porous support layer [24], which was subsequently stored in DI water after it was removed from the water bath till later usage, as shown in Figure 1.

#### 2.2.2. The Preparation of the Modified Active Selective Layer

**Figure 2:**
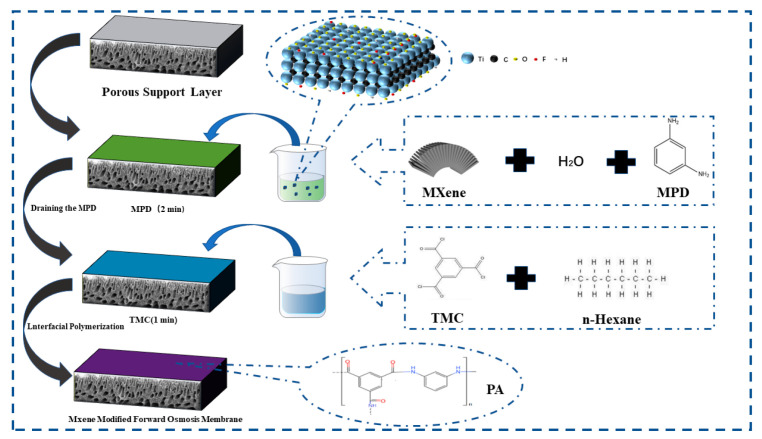
The active selective layer preparation by the interfacial polymerization reaction.

The active selective layer was synthesized via classical interfacial polymerization [25]. Firstly, the MXene nano-sheets were dissolved in pure water by ultrasonic for 30 min, followed by stirring for 3 h to obtain the MXene nano-sheets dispersion. The MXene nano-sheets dispersion was mixed with MPD to form the liquid mixture, which was added to the top surface of the porous support layer, after which the substrate was held horizontally for 4 min. Next, the aqueous mixture was drained off, and the TMC/n-hexane solution was poured onto the porous support layer surface and drained off after 1 min of immersion. Finally, those as-prepared membranes were put into the oven for 5 min. The addition amounts of MXene nano-sheet of 0%, 0.005%, 0.007%, 0.01%, and 0.03%, respectively, correspond to the prepared FO membrane numbers T-1, T-2, T-3, T-4, and T-5, as shown in Figure 2.

### 2.3. The Characterization of MXene Nano-Sheets Modified FO Membrane

The ATR mode transformed infrared spectrum of the spectrometer (Thermo Fisher Nicolet, Chengdu, China) was used to analyze the functional groups of the modified FO membrane. The water contact angle of the membrane surface was measured by the contact angle meter (CAM, Kruss DSA100, Hamburg, Germany). The surface morphology and surface roughness of the modified FO membrane were measured by an emission scanning electron microscope (FEI inspect F50 FSEM, Hillsboro, OR, USA) and an atomic force microscope (dimension icon AFM, Billerica, MA, USA) respectively. The surface chemical characterization and crosslinking degree of the active selective layer of the FO membrane were tested by X-ray electron spectroscopy (XPS, Thermo Scientific Escalab250 Xi, Waltham, MA, USA). 

### 2.4. The Permeability Test of the MXene Nano-Sheets Modified FO Membrane

#### 2.4.1. Test Equipment

**Figure 3:**
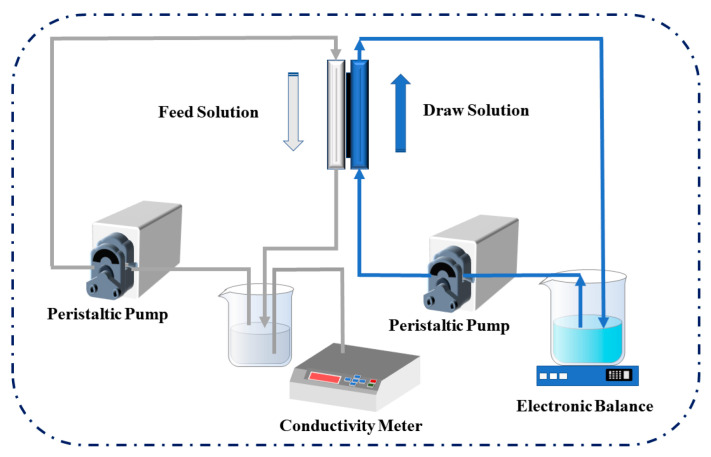
The schematic diagram of the FO performance testing system.

The FO membrane test equipment mainly included peristaltic pumps (Jie Heng, Chongqing, China), an electronic balance (APT456, Shenzhen, China), and a self-made membrane module (the effective area was 8.0 cm^2^) [26]. Because the FO membrane was asymmetric, different membrane orientations had a great impact on the results. Therefore, the membrane permeability and separation performance under the two modes of the active selective layer facing the draw solution (AL-DS mode) and the active layer facing the feed solution (AL-FS mode) were measured accordingly. The 1 M NaCl solution and pure water were selected as the draw solution and feed solution. As shown in Figure 3, the change in the weight of the draw solution during the FO process was recorded by the balance and the change in the conductivity on the feed solution side was recorded by the conductivity meter (Lei Ci, Shanghai, China).

#### 2.4.2. Test Parameters

Water flux (*J_w_*), reverse solute flux (*J_s_*), and specific reverse solute flux (*F_S_*) were used as FO membrane test indicators [27]. The higher the water flux, the better the permeability of the membrane. The interception performance of the membrane depends on the reverse solute flux. The smaller the reverse solute flux, the better the interception effect of the membrane. The specific reverse solute flux was a comprehensive index to measure the selectivity of the membrane, and the lower its value, the better the overall separation performance of the membrane.

The calculation formulas are:

$$J_{w}=\frac{\Delta m}{\rho \cdot t\cdot s}$$

where *J_w_* is the water flux of FO process (unit: L/m^2^·h), ∆*m* is the weight change in the draw solution (unit: g), ρ is the density of water (unit: g/m^3^), *t* is the operation time of FO process (unit: h), and s is the effective area of FO membrane module (unit: m^2^).

$$J_{s}=\frac{c\cdot m}{\rho \cdot t\cdot s}$$

where *J_s_* is the reverse solute flux of FO process (unit: g/m^2^·h), *m* is the weight of the feed solution after a certain time (unit: g), *c* is the concentration in the feed solution after a certain time (unit: g/L), ρ is the density of water (unit: g/m^3^), *t* is the operation time of FO process (unit: h), and s is the effective area of FO membrane module (unit:m^2^).

$$F_{S}=\frac{Js}{Jw}$$

where *F_S_* is the specific reverse solute flux of the FO process (unit: g/L).

## 3. Results and Discussion

### 3.1. The SEM Characterization of the MXene Nano-Sheets Modified FO Membrane

**Figure 4:**
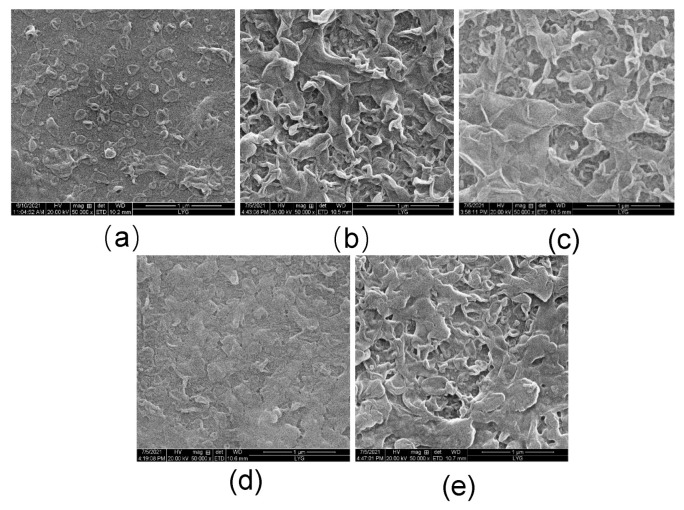
The SEM of the FO membranes with different amounts of MXene nano-sheet: (**a**) T-1, (**b**) T-2, (**c**) T-3, (**d**) T-4, and (**e**) T-5, respectively.

Figure 4 shows the SEM figure of the active selective layer of the FO membrane prepared with different MXene nano-sheet additions. It can be seen from the figure that all membrane surfaces showed the typical morphology of the active selective layer, demonstrating the structural characteristics of “synapse” and “sheet”, which also proved that the active selective layer was successfully prepared on the porous support layer through the interfacial polymerization reaction [28,29]. At the same time, it can also be seen that there were obvious differences in the prepared active selective layer on the FO surface because of the different addition amounts of MXene nano-sheet in the FO active selective layer, which indicates that the addition of different amounts of MXene nano-sheet can affect the structure of the active selective layer. When MXene nano-sheets were not added, due to the limitation of the membrane structure of the porous support layer, less aqueous phase solution was attached and the interfacial polymerization reaction rate was slow, the morphology and structural characteristics of the “synapse” of the polyamide active selective layer were not apparent. Therefore, there were fewer and insignificant “synapses” in the active selective layer of the T-1 membrane structure. When MXene nano-sheets were added to the aqueous phase, the structure of the active selective layer of the prepared FO membrane changed immediately, showing more “synapse” and a more conspicuous “flakes” structure. With the increase of MXene nano-sheet addition, more MXene nano-sheets participated in interfacial polymerization, which has a greater impact on the reaction rate of the aqueous phase and organic phase. Finally, due to the agglomeration of excessive MXene nano-sheets in the active selective layer [30], the polyamide structure was affected, and the “synapse” and “sheet” structures were significantly reduced, so the structural characteristics of the active selective layer from T-3 to T-5 membrane became gentle and dense [31].

### 3.2. The Atomic Force Microscopy Characterization of the MXene Nano-Sheets Modified FO Membrane

**Figure 5:**
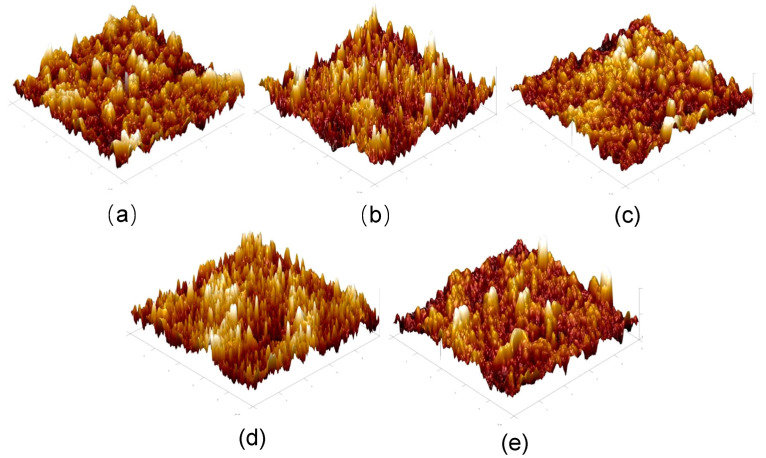
The AFM images of the FO membrane with different amounts of MXene nano-sheet: (**a**) T-1, (**b**) T-2, (**c**) T-3, (**d**) T-4, and (**e**) T-5, respectively.

Figure 5 shows the AFM images of the active selective layer of the FO membrane modified by MXene nano-sheets. By observing the morphology in the figure, it can be seen that all the membrane surfaces showed a typical polyamide “valley ridge” structure, which also showed the success of preparing an active selective layer by the interfacial polymerization reaction again. In addition, it can be seen that with the gradual increase of the addition amount of MXene nano-sheets, the protrusion morphology in this figure indicated the principle of first sharp and then gentle, which meant that MXene nano-sheets had a severe impact on the reaction process of interfacial polymerization after being adding into the aqueous phase. Therefore, MXene nano-sheet incorporation had a great impact on the formation of the membrane structure and morphological changes.

The surface roughness of the active selective layer corresponding to the FO membrane was modified by the addition of the different MXene nano-sheets in Table 1. With the addition of MXene nano-sheets, the surface roughness of the active selective layer underwent a special change. The principle shown in this table was that with the addition of MXene nano-sheets, the roughness of the surface-active selective layer first rose suddenly, then decreased gradually, and finally decreased slightly. From the point of view of the interfacial polymerization reaction, it can be explained that the diffusion of the aqueous phase usually leads to the formation of a ridge structure. That was the formation of the “ridge” shape, which improved the surface roughness of the active selective layer. When MXene nano-sheets were added in small amounts, they not only inhibited aqueous phase diffusion into the organic phase, but they also promoted aqueous phase-organic phase reaction.Therefore, it facilitated the formation of a “ridge” structure, and the surface roughness increased suddenly. When MXene nano-sheets were added continuously (0.007–0.01%) at this time, the content of MXene nano-sheets dispersed in the aqueous phase increased, which hindered the diffusion from the aqueous phase to the organic phase, limiting the degree of interfacial polymerization and forming a smoother membrane surface, the roughness then decreased [32]. In addition, the excessive MXene nano-sheet content (0.03%) did not alleviate the agglomeration phenomenon induced inhibition of aqueous phase diffusion, but slightly increased membrane surface roughness due to large MXene nano-sheet aggregation and destruction of the membrane surface structure.

### 3.3. The Fourier Transform Infrared Spectroscopy Characterization of the MXene Nano-Sheets Modified FO Membrane

**Figure 6:**
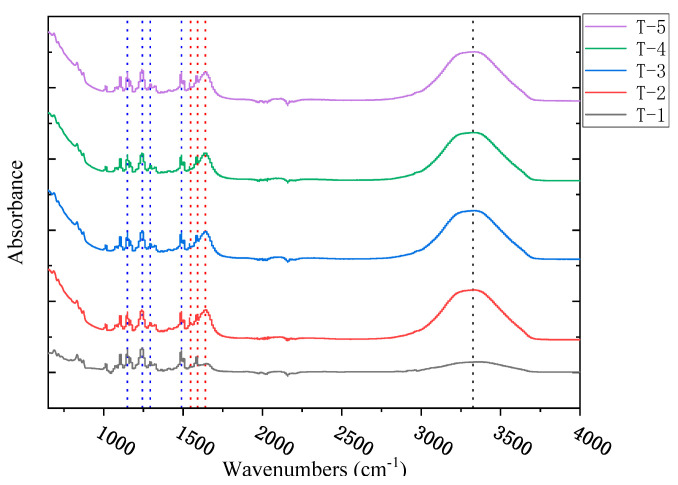
The ATR-FTIR spectra of the FO membrane with different amounts of MXene nano-sheet.

Figure 6 shows the test results of the Fourier infrared spectra of T-1 to T-5 membranes. It can be seen from the figure that there were a series of characteristic peaks, such as the symmetrical O=S=O peak (1148 cm^−1^), the symmetrical C-O-C peak (1240 cm^−1^), the asymmetric O=S=O peak (1295 cm^−1^), and the CH_3_-C-CH_3_ (1500 cm^−1^) peak of the PSf porous support membrane, indicating the smooth synthesis of a porous support layer. The obvious characteristic peaks were also observed at about 1600 cm^−1^, 1650 cm^−1^ and 1545 cm^−1^, which were attributed to the aromatic ring, amide I band (C=O), and amide II band (C-H) in the active selective layer, respectively. This confirmed again that the surface-active selective layer was successfully prepared on the surface of the porous support layer. In addition, in the range of 2000–4000 cm^−1^, it can be seen that the characteristic peak intensity of about 3300 cm^−1^ was significantly enhanced, which was due to the O-H bond vibration, indicating that the addition of MXene nano-sheets helped the FO surface form a more hydrophilic active selective layer [33].

### 3.4. The X-ray Photoelectron Spectroscopy Characterization of The MXene Nano-Sheets Modified FO Membrane

**Figure 7:**
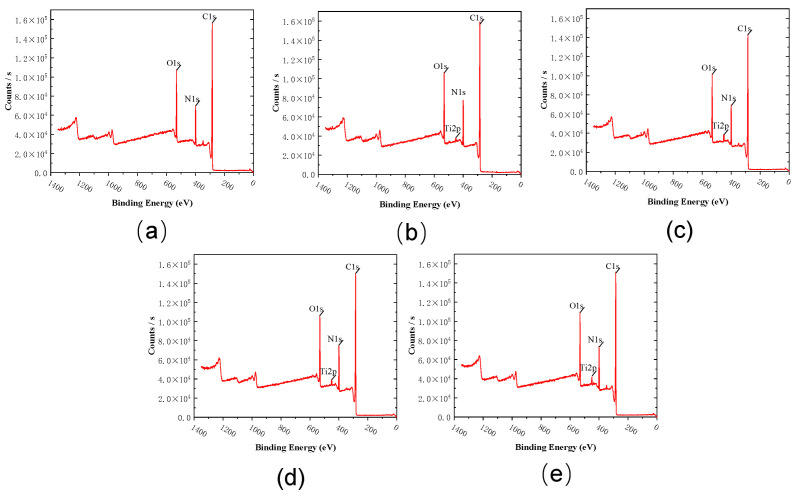
The XPS spectra of the surface of the FO membranes with different amounts of MXene nano-sheet: (**a**) T-1, (**b**) T-2, (**c**) T-3, (**d**) T-4, and (**e**) T-5, respectively.

Figure 7 shows the XPS test results of the prepared MXene nano-sheets modified FO membrane. It can be seen that the elements C, N, and O appear in T-1 to T-5 membranes. Furthermore, the elements Ti appear in diagrams T-2 to T-5, indicating that MXene nano-sheets were successfully added to the polyamide active selective layer. According to Table 2, we can know the percentage content of C, O, and N and calculate the cross-linking degree of the FO membrane surface by calculating the O/N ratio. We can analyze the O/N element ratio in the active selective layer of the FO membrane surface to know the cross-linking degree of the membrane surface. The influence trend of MXene nano-sheets on the crosslinking reaction of the polyamide active selective layer can be inferred from the relationship between the O/N ratio and crosslinking degree. The reason was that the lower the O/N value, the higher the cross-linking degree of the membrane surface, the more favorable the salt interception of the active selective layer on the membrane surface. The higher the O/N value, the lower the cross-linking degree of the membrane surface, which was not conducive to the interception of salt by the membrane surface active selective layer [34]. The XPS results showed that the addition of MXene nano-sheets can affect the oxygen-nitrogen ratio and change the crosslinking degree. It can be seen from the table that the O/N value of the membrane surface active selective layer decreased, then increased and finally decreased with the addition of MXene nano-sheets. The change results demonstrated that the method of adding MXene nano-sheets to the aqueous phase can significantly accelerate or inhibit the formation of the surface-active selective layer. It indicated that the MXene nano-sheets affected the diffusion rate between the aqueous phase and organic phase and directly influenced the cross-linking reaction between the aqueous phase and organic phase. When a small amount of MXene nano-sheets was added, it had a positive effect on the interfacial polymerization reaction, promoting the full reaction between the aqueous and organic phases and improving the crosslinking degree. However, when MXene nano-sheets were continuously added (0.007–0.01%), the oxygen-nitrogen ratio increased and the crosslinking degree decreased, which may be because the MXene nano-sheets hindered the diffusion from the aqueous phase to the organic phase and inhibited the crosslinking reaction [35]. Continuing to increase the content of MXene nano-sheets (0.03%), at this time, the oxygen-nitrogen ratio decreased slightly and the crosslinking degree increased slightly because the excessive addition of MXene nano-sheets led to agglomeration, reducing the inhibition of diffusion from aqueous phase to organic phase, and the interfacial polymerization reaction was relatively slightly strengthened [36].

### 3.5. The Characterization of Hydrophilicity of the MXene Nano-Sheets Modified FO Membrane

**Figure 8:**
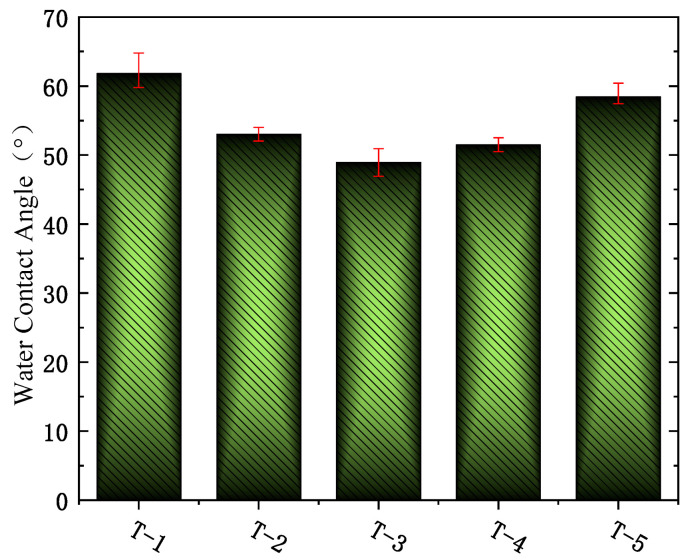
The water contact angles of the FO membranes with different amounts of MXene nano-sheet.

Figure 8 shows the effects of different MXene nano-sheets’ additions on the water contact angle of the FO membrane. It can be seen from the figure that the water contact angles of T-1 to T-5 membrane surfaces were 61.7°, 53.02°, 48.92°, 51.49°, and 58.43°, respectively. Due to the hydrophilic characteristics of MXene nano-sheets, they had an obvious positive effect on the improvement of membrane surface hydrophilicity. With the addition of MXene nano-sheets, the water contact angle of the surface-active selective layer of the FO membrane decreased first and then increased, and its water contact angle was lower than that of the unmodified FO membrane, which showed that the FO membrane generated a more hydrophilic surface due to the addition of MXene nano-sheets. On the one hand, the MXene nano-sheets contained a large number of O-H bonds of hydrophilic groups. Through the method of introducing them into the aqueous phase, the MXene nano-sheets can directly participate in the crosslinking reaction between the aqueous phase and the organic phase, thus affecting the polyamide structure of the active selective layer to the greatest extent, generating a more hydrophilic surface-active selective layer and improving the hydrophilicity of the prepared FO membrane. On the other hand, a large number of hydrophilic functional groups (C=O and N-H) rich in MXene nano-sheets were directly attached to the surface of the active selective layer, making the surface of the FO membrane highly hydrophilic [37]. However, with the continuous increase of MXene nanosheets, the contact angle increased and the hydrophilicity decreased in the figure, which may be due to the agglomeration of excessive MXene nano-sheets, resulting in the reduction of hydrophilic groups and the destruction of the surface structure of the active selective layer.

### 3.6. The Permeability Test of the MXene Nano-Sheets Modified FO Membrane

It can be seen from the above that the FO membrane constructed by inserting MXene nano-sheets into the aqueous phase had apparent distinctive changes in physical structure, chemical content, and surface shape. To further examine the impact of these alterations on the permeation separation performance of the FO membrane, we tested the osmotic separation performance of the FO membranes modified with varied MXene nano-sheet content.

#### 3.6.1. The Effects of MXene Nano-Sheets on Water Flux of the FO Membrane

**Figure 9:**
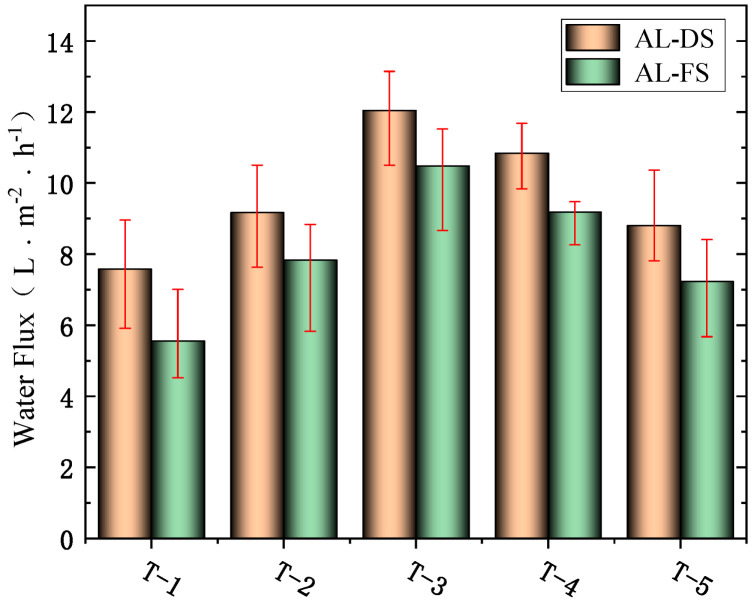
The effects of different amounts of MXene nano-sheet on water flux of the FO membrane.

It can be seen from Figure 9 that the water flux of FO increased first and then decreased with the increase of MXene nano-sheet content; when MXene nano-sheets were not added, the water flux of the prepared FO membrane was 7.58 L·m^−2^·h^−1^ (AL-DS mode) and 5.56 L·m^−2^·h^−1^ (AL-FS mode). When the addition amount was 0.005%, the water flux of the prepared FO membrane was 9.17 L·m^−2^·h^−1^ (AL-DS mode) and 7.83 L·m^−2^·h^−1^ (AL-FS mode). When the addition amount was 0.007%, the water flux of the prepared FO membrane was 12.05 L·m^−2^·h^−1^ (AL-DS mode) and 10.49 L·m^−2^·h^−1^ (AL-FS mode). At this time, the addition of MXene nano-sheets showed a positive correlation with the water flux, and then the water flux began to decline. When the MXene nano-sheets were added at 0.01%, the water flux of the prepared FO membrane decreased to 10.84 L·m^−2^·h^−1^ (AL-DS mode) and 9.19 L·m^−2^·h^−1^ (AL-FS mode). When MXene nano-sheets were added at 0.03%, the water flux of the prepared FO membrane continued to decrease to 8.81 L·m^−2^·h^−1^ (AL-DS mode) and 7.23 L·m^−2^·h^−1^ (AL-FS mode). It can be seen that the water flux of FO membrane modified by MXene nano-sheets was higher than that of unmodified FO membrane, and when the addition amount of MXene nano-sheets was 0.007%, the highest water flux reached 12.05 L·m^−2^·h^−1^ (AL-DS mode) and 10.49 L·m^−2^·h^−1^ (AL-FS mode). According to the above physical and chemical characterization, when the addition amount of MXene nano-sheets was within a certain range (0.005–0.007%), the MXene nano-sheets were evenly dispersed in the active selective layer. Although they inhibited the cross-linking reaction and reduced the roughness, the MXene nano-sheets were rich in a large number of hydrophilic groups, and this greatly enhanced the hydrophilicity of the active selective layer. In addition, because the MXene nano-sheets were directly distributed in the active selective layer, they had special transmission channels and could directly transport a large number of water molecules, which eventually led to an increase in water flux [38]. However, when the amount of MXene nano-sheets was too high (0.01–0.03%), the aggregation phenomenon occurred in the membrane due to the high concentration. When introducing nanoparticles, this phenomenon was very common, and some researchers developed catalysts to alleviate nanoparticle agglomeration [39,40], which can be used as a strategy to further optimize the modified membrane of MXene nano-sheets. At the same time, according to the contact angle test above, the hydrophilicity also decreased to a certain extent, and the water channel was blocked or reduced, which also destroyed the surface structure of the active selective layer so that the water flux finally decreased synchronously.

#### 3.6.2. The Effects of MXene Nano-Sheets on the Reverse Solute Flux of FO Membrane

**Figure 10:**
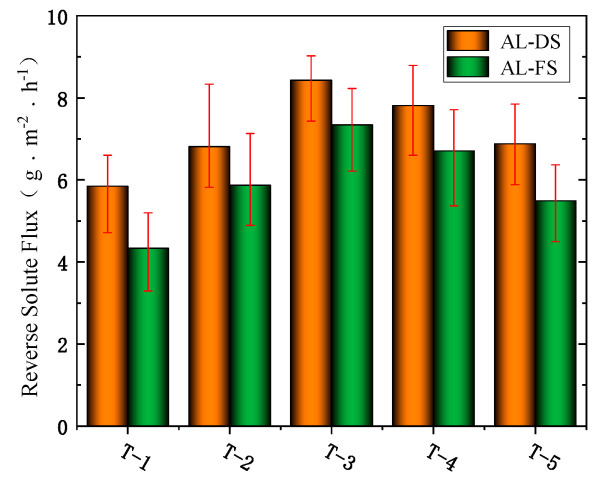
The effects of different amounts of MXene nano-sheet on the reverse solute flux of the FO membrane.

Figure 10 shows that as the amount of MXene nano-sheet added increased, the reverse solute flux of the modified FO membrane increased and then decreased slightly. When no MXene nano-sheets were added, the reverse solute flux of the prepared FO membrane was 5.85 g·m^−2^·h^−1^ (AL-DS mode) and 4.33 g·m^−2^·h^−1^ (AL-FS mode), and when the addition amount was 0.005%, the reverse solute flux of the prepared FO membrane was 6.81 g·m^−2^·h^−1^ (AL-DS mode) and 5.87 g·m^−2^·h^−1^ (AL-FS mode). At this moment, it shows an upward trend. When the addition amount was 0.007%, the reverse solute flux of the prepared FO membrane was 8.43 g·m^−2^·h^−1^ (AL-DS mode) and 7.34 g·m^−2^·h^−1^ (AL-FS mode), and it reached the maximum of these groups of experiments. When the addition amount continued to increase to 0.01%, the reverse solute flux of the prepared FO membrane was 7.81 g·m^−2^·h^−1^ (AL-DS mode) and 6.71 g·m^−2^·h^−1^ (AL-FS mode). At this time, the reverse solute flux showed a slight downward trend. When the addition amount reached 0.03%, the reverse solute flux of the prepared FO membrane decreased to 6.88 g·m^−2^·h^−1^ (AL-DS mode) and 5.49 g·m^−2^·h^−1^ (AL-FS mode) finally. It can be seen that the reverse solute flux of the FO membrane modified by MXene nano-sheets was higher than that of the unmodified FO membrane. The highest reverse solute flux reached 8.43 g·m^−2^·h^−1^ (AL-DS mode) and 7.34 g·m^−2^·h^−1^ (AL-FS mode) when the addition of MXene nano-sheets was 0.007%. The addition of MXene nano-sheets also changed the membrane structure of the active selective layer. The MXene nano-sheets added to the aqueous phase can increase the reverse solute flux, and the reverse solute flux of the modified FO membrane was slightly higher than that of the unmodified FO membrane. According to the permeability of the FO membrane and the above physical and chemical characterization, this was mainly due to the swelling effect produced by the MXene nano-sheets themselves [41,42]. When the Na^+^ ions in the draw solution pass through the channel formed by the MXene nano-sheets, the Na^+^ ions were quickly intercalated into the MXene nano-sheets layer. At the same time, with the electrostatic interaction between Na^+^ ions and negatively charged MXene nano-sheets, the layer spacing of MXene nano-sheets was expanded and the formed nanochannel was widened. As a result, Na^+^ ions can pass through quickly without obstruction. Moreover, Na^+^ ions can directly enter the active selective layer and make contact with MXene nano-sheets, and the swelling effect was more obvious because the MXene nano-sheets were distributed in the active selective layer, so the increase in reverse solute flux was greater. When the MXene nano-sheets continued to be added (0.01−0.03%), at that time, due to the agglomeration of some MXene nano-sheets and the reduction of nano-channels, the swelling effect tended to be gentle, and the membrane pores in the active selective layer were blocked, so the reverse solute flux showed a downward trend.

#### 3.6.3. The Specific Reverse Solute Flux

**Figure 11:**
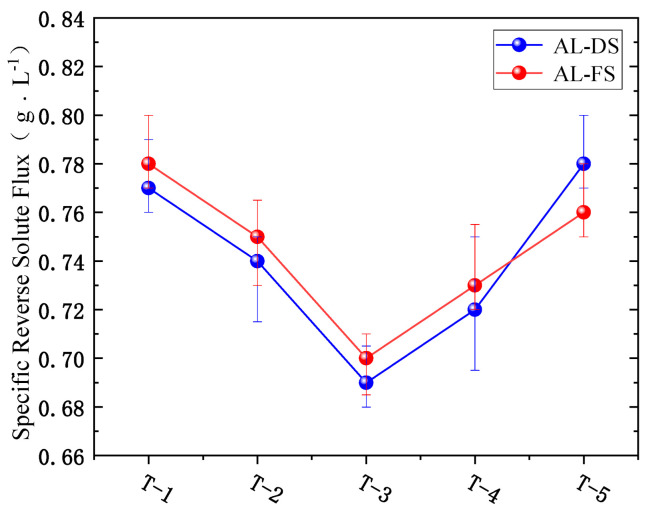
The effects of different amounts of MXene nano-sheet on the specific reverse solute flux of the FO membrane.

Figure 11 shows the ratio of water flux and reverse solute flux under two different modes (AL-DS and AL-FS). That was the specific reverse solute flux, which can truly reflect the brine separation performance of an FO membrane. As shown in the figure, the trend of specific reverse solute flux in AL-DS mode and AL-FS mode was consistent. With the gradual increase of MXene nano-sheets addition, the specific reverse solute flux of the FO membrane first decreased and then increased: in AL-DS mode, it was 0.74 when the addition was 0.005%, 0.69 when the addition was 0.007%, and 0.72 when the addition was 0.01%. When the addition amount was 0.03%, it was 0.78. In AL-FS mode, it was 0.75 when the addition amount was 0.005%, 0.70 when the addition amount was 0.007%, 0.73 when the addition amount was 0.01%, and 0.76 when the addition amount was 0.03%. According to the comparison of the above results, when the addition amount was 0.007%, the membrane separation performance of the FO membrane was the best among those resultant membranes.

## 4. Conclusions

In this paper, we investigated a modified FO membrane using MXene nano-sheets in the interfacial polymerization process. The following conclusions were drawn from comparing the surface morphology, roughness, chemical structure, hydrophilicity, and osmotic separation performance of the modified FO membrane with different addition amounts of MXene nano-sheet: The active selective layer of the FO membrane lost synaptic structure and surface morphology as the concentration of MXene nano-sheets in the aqueous phase increased. Adding MXene nano-sheets reduced the O/N ratio, improved the crosslinking degree of the active selective layer, and increased the hydrophilicity of the produced FO membrane. The water flux and reverse solute flux of the FO membrane modified by MXene nano-sheets increased first, then reduced in the AL-DS and AL-FS modes. The water flux increased greatly, but the reverse solute flux increased only slightly, and the membrane separation performance measuring parameter specific reverse solute flux was optimized. This shows that MXene nano-sheets might improve the performance of FO membranes and have potential applications in membrane separation.

## Figures and Tables

**Table 1:** The surface roughness of the FO membranes with different amounts of MXene nano-sheet.

FO Membrane	Ra (nm)	Rms (nm)
T-1	23.9	34.6
T-2	33.2	42.5
T-3	30.1	39.5
T-4	26.6	35.1
T-5	31.4	39.6

**Table 2:** The elements composition and O/N ratios of the FO membrane with different amounts of MXene nano-sheet.

FO Membrane	C Content (%)	O Content (%)	N Content (%)	O/N Value
T-1	74.04	14.69	11.27	1.30
T-2	73.72	14.42	11.55	1.24
T-3	73.68	14.19	11.16	1.27
T-4	73.92	14.29	11.08	1.29
T-5	73.11	15.01	11.69	1.28

## Data Availability

Not applicable.
